# Deep Panning: Steps towards Probing the IgOme

**DOI:** 10.1371/journal.pone.0041469

**Published:** 2012-08-01

**Authors:** Arie Ryvkin, Haim Ashkenazy, Larisa Smelyanski, Gilad Kaplan, Osnat Penn, Yael Weiss-Ottolenghi, Eyal Privman, Peter B. Ngam, James E. Woodward, Gregory D. May, Callum Bell, Tal Pupko, Jonathan M. Gershoni

**Affiliations:** 1 Department of Cell Research and Immunology, Tel Aviv University, Tel Aviv, Israel; 2 National Center for Genome Resources, Santa Fe, New Mexico, United States of America; Istituto Superiore di Sanità, Italy

## Abstract

**Background:**

Polyclonal serum consists of vast collections of antibodies, products of differentiated B-cells. The spectrum of antibody specificities is dynamic and varies with age, physiology, and exposure to pathological insults. The complete repertoire of antibody specificities in blood, the IgOme, is therefore an extraordinarily rich source of information–a molecular record of previous encounters as well as a status report of current immune activity. The ability to profile antibody specificities of polyclonal serum at exceptionally high resolution has been an important and serious challenge which can now be overcome.

**Methodology/Principal Findings:**

Here we illustrate the application of Deep Panning, a method that combines the flexibility of combinatorial phage display of random peptides with the power of high-throughput deep sequencing. Deep Panning is first applied to evaluate the quality and diversity of naïve random peptide libraries. The production of very large data sets, hundreds of thousands of peptides, has revealed unexpected properties of combinatorial random peptide libraries and indicates correctives to ensure the quality of the libraries generated. Next, Deep Panning is used to analyze a model monoclonal antibody in addition to allowing one to follow the dynamics of biopanning and peptide selection. Finally Deep Panning is applied to profile polyclonal sera derived from HIV infected individuals.

**Conclusions/Significance:**

The ability to generate and characterize hundreds of thousands of affinity-selected peptides creates an effective means towards the interrogation of the IgOme and understanding of the humoral response to disease. Deep Panning should open the door to new possibilities for serological diagnostics, vaccine design and the discovery of the correlates of immunity to emerging infectious agents.

## Introduction

Polyclonal serum consists of vast collections of antibodies, the products of differentiated B-cells [1], [2]. The B-cell repertoire can be divided into three categories: potential, available and utilized [1]. The total “potential B-cell repertoire” is derived from the combinatorial product of the VDJ and VJ germ-line genes amplified by the effect of junctional P and N nucleotides plus somatic hyper mutations, leading to values as high as 10^11^ unique molecules [3]. This number, however, supersedes the total amount of B-cells in a person and thus one should consider the “available B-cell repertoire” - the actual clonal diversity of B-cells that exists in an individual (estimated to be at least 1.6×10^5^ for the light chain and in the range of 2–20×10^5^ for the heavy chain [3], [4] where some estimates [5] are as high as 9×10^6^, for review see [6]). Naïve B-cells, go on to differentiate into antibody secreting cells (ASC) and memory cells upon encounter with antigens recognized by their cell surface B-cell receptor (BCR) [7], [8]. Therefore, the observed diversity of antibodies present in serum corresponds to the “utilized B-cell repertoire”; those B-cells of the available repertoire that have been stimulated to produce ASCs [2], [9]. The spectrum of antibody specificities is dynamic and varies with age, physiological status and exposure to pathological insults [2], [7], [10], [11]. The complete repertoire of antibody specificities in blood, the IgOme, is therefore an extraordinarily rich source of information – a molecular record of previous encounters as well as a status report of current immune activity [12], [13], [14], [15].

Antibodies provide a first line of defense, detecting invading pathogens, neutralizing and clearing them. The surveillance and response towards emerging malignancies relies on antibodies as well. Hence, measuring antibody specificity is fundamental to sero-diagnosis. Moreover, comprehensive analysis of the activities of serum antibodies provides insights to vaccine design as well as the ability to evaluate vaccine efficacy [16], [17], [18], [19], [20].

Here we focus on how to profile the diversities of antibody binding activities of serum. For this we combine the flexibility of combinatorial phage display with the power of high throughput deep sequencing leading to “Deep Panning” – a means towards interrogating the IgOme.

### Random Peptides as Probes of Antibody Specificity

Phage display is widely employed in the production of random peptide libraries used to survey the universe of antibody specificities [20], [21]. Screening random peptide libraries generates defining panels of the diversity of peptides that are affinity selected by the specific antibodies used as bait. Whereas initially expression via Protein 3 was the first mode used to display random peptides on filamentous bacteriophages [22], applications an alternative system, display via the phage’s major coat protein - Protein 8, produces highly polyvalent phages that often improves the analysis and sensitivity of antibody-peptide binding [23].

Some 2,700 copies of Protein 8 encapsidate the entire length of the viral ssDNA. Genetic alteration of the phages’ single *protein 8* gene would lead to a phage homogenously modified along its entire shaft as all copies of the Protein 8 would contain the foreign insert. This however, could be problematic as inserts exceeding 6–8 residues in length interfere with the packing of the Protein 8 into the growing filament and would thus disrupt phage assembly [24], [25]. This obstacle is routinely circumvented when expression of longer Protein 8 fusions is performed by using two functional *protein 8* genes; one expressing the wild type Protein 8 and the other the recombinant Protein 8 containing the foreign peptide. As a result “chimeric phages” are produced where most Protein 8’s are wild type, interspersed with copies (tens to hundreds) of recombinant Protein 8 [26]. Affinity selection of peptides/phages is achieved via an antibody pull-down process coined “Bio-panning”.

Bio-panning is in essence an affinity capture experiment where phages displaying peptides that are recognized by the antibody are bound and separated from those that are not. Typically, panels of tens of affinity selected peptides are cloned and sequenced to provide a dataset from which information pertaining to antibody specificity can be extracted. When one bio-pans a monoclonal antibody (mAb), tens of peptides may suffice to portray the binding characteristics and motifs recognized by the mAb. However, the complexity of antibodies in serum is extensive where each antibody may bind numerous different peptides, each antigen stimulating the production of multiple antibodies and each pathogen being comprised of many antigens. Clearly tens of affinity selected peptides cannot provide a comprehensive depiction of the IgOme. By employing next generation deep sequencing as a reader of affinity selected phage displayed peptides, Deep Panning generates comprehensive and high resolution profiles of the antibodies in serum.

## Results

### Adapting Phage Display to Deep Sequencing

**Figure 1:**
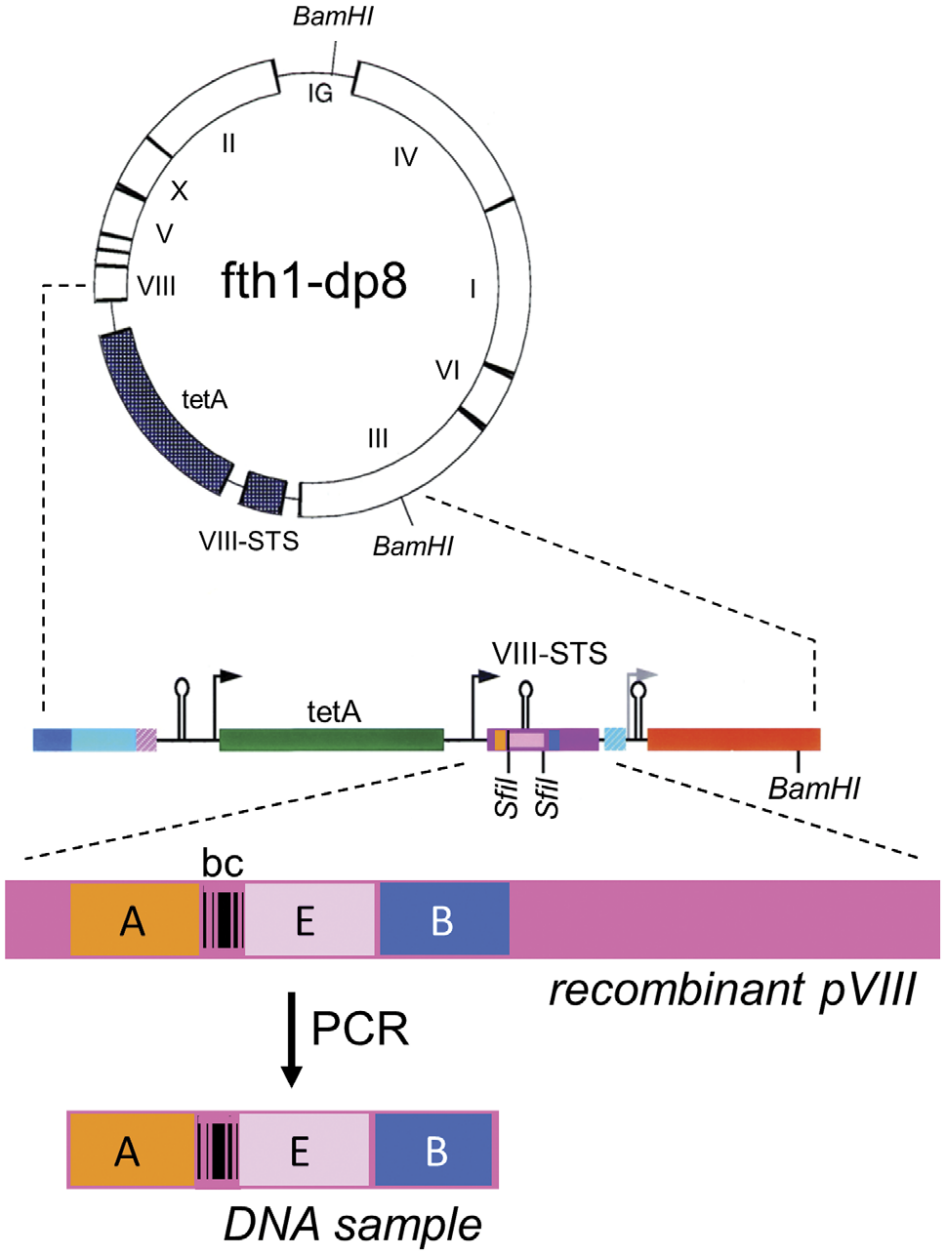
The fth1-dp8 vector. The recombinant *protein 8* gene of the fth1 vector (VIII-STS) was modified by introducing the 5′ (A – orange) and 3′ (B – blue) Illumina adaptors such that they flank the DNA insert (E). Reference DNA barcodes were introduced between the A adaptor and the first SfiI site (bc, see **“**Pre-processing of sequence data” in Methods). Samples for deep sequencing are generated directly by PCR using the adaptors as PCR primers.

Over the past decade we have used the chimeric phage system producing libraries of random peptides ranging in length from 6 to 12 amino acids, with and without flanking disulfides to produce constrained looped peptides. In order to dramatically increase the peptide database in such experiments we adapted our Protein 8 phage display system to next generation high throughput sequencing [27]. For most next generation deep sequencing methods the target DNA to be sequenced is flanked by adaptor sequences compatible with the specific system being used. As is illustrated in Figure 1 we modified the fth1 phage display vector [28] to contain the Illumina adaptor sequences [29] upstream and downstream to the SfiI cloning sites of the recombinant *protein 8* gene thus generating fth1-dp. The DNA adaptors were selected so to generate compatible peptide compositions and avoid stop codons.

To test the fth1-dp system, we cloned oligonucleotides corresponding to the linear peptide epitope of the murine mAb GV4H3 (221-AGFAIL-226) derived from HIV-1 gp120 [30] into the modified fth1-dp vector. The chimeric phages successfully displayed the insert and were selectively bound by the mAb indicating that the recombinant Protein 8 was assembled and amenable to affinity selection (Figure S1).

### How Random are Peptide Libraries?

**Figure 2:**
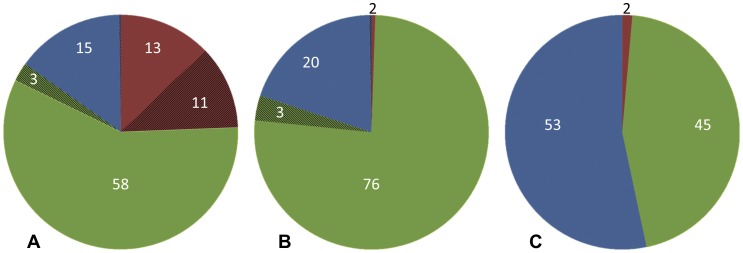
Pie charts depicting the proportion of unique peptides in phage display libraries. A total of 155,241 inserts were read for the random phage display peptide library (**A**). 24% of the peptides contained at least one UAA or UGA stop codon (red plus dark red). 58% of the peptides were unique containing a UAG stop codon (light green) of these some exist in multiple copies (3% of the total, dark green). 15% of the peptides were completely devoid of stop codons (blue, less than 1% had 2–5 copies). Pie Chart (**B**) depicts the same set of peptides devoid of all those that had detectable frameshifted inserts (37,223 inserts leaving 118,018 functional peptides of which ca 1% contained stop codons UAA and UGA nonetheless). A second library was constructed in DH5alpha *supE144* cells (**C**). Values below 1% are not given.

As a case in point,using the fth1-dp vector we constructed a random peptide library consisting of a total of 2×10^9^ random 7 mer linear peptides (NNK codons were used to avoid UAA and UGA stop codons). A sample of the library was subjected to direct PCR amplification using primers corresponding to the upstream and downstream adaptor sequences thus generating amplified DNA segments directly ready for Illumina deep sequencing. The PCR product was quantified and a small quantity was added to the phi-X control channel of an Illumina GAIIx DNA sequencer (“single read” mode of 54 bases). A total of 155,241 DNA sequences were obtained of which 132,887 were unique (Figure 2A). Translating the inserts was revealing as it turned out that >37,000 sequences contained UAA and UGA stop codons leading to Protein 8 truncation and thus generating phenotypically wild-type phages. This situation is curious as NNK prohibits an A in the third position of the codon. The vast majority of these UAA/UGA containing phages turn out to be aberrations and result from dysfunctional oligonucleotide insertions leading to detected frame shifts distorting the intended reading frame. Of these stop codon containing phages, only half were unique; the rest appeared in multiple copies where the most prevalent insert was found 17,044 times. This indicates that truncation of recombinant Protein 8 provides a selective advantage; phage assembly and incorporation of recombinant Protein 8 appear to be more demanding than that of wild-type Protein 8. A corrected pie-chart is given in Figure 2B, in which all the frame shifted inserts were removed (note a remaining 1% of the inserts continue to contain UAA and UGA stop codons with no apparent frame shift in the 54 base read).

A second surprising observation was that 79% of the non-frame shifted inserts contained at least one UAG stop codon. The theoretical expected frequency of UAG containing phages is about 20%. A selective advantage of UAG stop codon could be transiently realized during the initial construction of the library which is performed in MC1061 cells chosen for their high efficiency for electroporation. Thus for the first 24 hours of phage library preparation, UAG functions as a stop codon leading to the observed over representation of those phages that contain this codon in their recombinant Protein 8. The library is then amplified and maintained in DH5alpha cells that contain the *supE144* suppression gene translating UAG as glutamine and thus circumventing abortive termination and ensuring the production of functional recombinant Protein 8.

In order to test the hypothesis that the over-abundance of UAG containing phages was due to the lack of suppression in MC1061 cells, another library was constructed, however this time the initial electroporation was performed using DH5alpha cells from the start. Although the transformation efficiency was markedly less, as expected (total complexity *ca* 10^8^phages), the profile of inserts was dramatically improved (Figure 2C). Fifty-three percent of the sequences had no stop codons and were virtually all unique. Forty-five percent of the library contained only UAG of the possible three stop codons, although the bias for UAG was not completely resolved and is currently further being investigated.

Next we turned to the copy number of the most prevalent peptides in each library and asked if such multiplicity could be a random event or rather indicative of peptides that have some selective advantage? For this we performed a simulation study, as described in the Methods section, in which we studied the distribution of the most common peptide in a naïve library, i.e., a library that was not exposed to any prior affinity selection. Our simulations showed that in all 100 simulations, the most frequent peptide never exceeded 4 copies. From these simulations we conclude that peptides that appear 4 or fewer times are expected by chance. For the library presented in Figure 2B, the top most frequent 38 unique peptides (205 total peptides) were in the range of 5–8 copies. This indicates that the number of copies for 99.99% of the peptides in this naïve library is as expected from a truly random library. We conclude that if some source of selection takes place in naïve libraries, it only affects a tiny portion of the peptides, and even these peptides are only amplified to a very limited extent. While this demonstrates relatively little bias towards common peptides, one must consider the fact that the profile of peptides expressed does have a distinct bias towards peptides containing glutamine (the result from the suppression of the over-represented UAG codon). In view of this, the following analyses were conducted using only peptides devoid of any stop codon.

### Following the Dynamics of Deep Panning

The composition of polyclonal serum is in essence a compound mixture of mAbs, each with its signature specificity embodied by the collection of peptides it binds. Thus it is anticipated that the spectrum of peptides recognized by the ensemble of antibodies of polyclonal serum will be extremely complex. Therefore, before embarking on the analysis of polyclonal serum, we first tested Deep Panning on a model mAb.

The murine mAb GV4H3 (mentioned above [30]) was used to pan 10^11^ phages of a 7 mer random peptide library to produce three samples: the first affinity capture (Capture #1) followed by two consecutive rounds of biopanning (amplification and capture, i.e., Captures #2 and #3). For each sample the captured phages were eluted and directly amplified by PCR. Each of these DNA samples (the PCR products) was added to the phi-X control lane of a GAIIx flow cell and the raw data were filtered to exclude DNA reads that would correspond to peptides containing any of the three stop codons. The 20 top most frequent peptides for each capture are given in Table 1 as well as the top peptides of the naïve library for comparison. It should be noted that the number of copies obtained for each peptide in the different samples simply reflects their relative concentration after random sampling of the eluted phages, PCR amplification and the fortuitous level of DNA used to spike the Illumina flow cell in each case. The total number of reads for each sample is given along with the number of unique peptides.

**Table 1:** Three rounds of panning with mAb GV4H3.

	naïve		Capture 1		Capture 2		Capture 3	
	sequence	copy number	sequence	copy number	sequence	copy number	sequence	copy number
1	RIRSEEL	24	**ADGIVGW**	3,105	**ADGIVGW**	11,489	**ADGIVGW**	65,003
2	TVVVAAG	17	**LAAGAVW**	3,039	**SAGFAME**	5,173	**SAGFAME**	6,630
3	VQLSIIV	15	**SAGFAME**	2,944	**LAAGAVW**	3,140	VGWAVLE	2,409
4	PRTTIMG	15	VSLCSSR	2,925	***VTPHTGF***	1,172	VTPHTGF	2,366
5	KFVVAFC	14	SVTDYVE	2,855	***GAHVAGG***	672	**LAAGAVW**	2,364
6	LAPREGA	13	RMGIRAL	2,659	***LTACTGF***	660	SAGVAME	2,242
7	LMTARWC	13	DDDGLDG	1,931	LGWAVLD	506	ADGIVGG	1,009
8	FWALATW	13	SAARVFM	1,723	GTASVGF	444	***EVGWAVH***	905
9	YCVGDGC	13	QLYGARE	1,716	***SLGWAVP***	355	WVGWAVQ	834
10	WVNAATC	12	NRSREMG	1,707	GVGWAVP	344	***LGWAVLD***	768
11	SRGVGVG	12	IVPACRG	1,646	***AVGWAVP***	336	***AVGWAVP***	536
12	RRRPGAV	12	RLVYVPS	1,335	GPGMALE	324	AGWAVLE	469
13	QLVRGRW	12	TGCSSIL	1,166	PASLVGF	300	***LVGWALS***	453
14	SVFIMLR	11	AFHSGLT	1,106	SAGWALP	293	***SLGWAVP***	445
15	VFMGGCR	11	CSWTLER	996	***EVGWAVH***	272	***GAHVAGG***	413
16	TSEGALR	11	GGGVGLL	912	LNSMVGF	226	***LTACTGF***	368
17	SSRSSGG	11	WHAQVGF	852	***VGWAVLE***	222	GVGFALD	362
18	GRLSADG	11	ERRMGSC	844	QMPNLGF	215	GVGFALE	349
19	RRAVRFM	11	SGVGATH	770	LVGWALS	202	RVSAEVW	346
20	GLCAEAC	10	RLRWWGR	738	SAGWALE	201	LAGWALD	337
sum		261 (0.1%)		34,969 (10%)		26,546 (41%)		88,608 (74%)
#	168,993 (92%)	183,451	32,301 (9%)	342,712	6,609 (10%)	64,316	4,823 (4%)	118,548
	**unique**	**total**	**unique**	**total**	**unique**	**total**	**unique**	**total**

GV4H3 mAb was used to bio-pan the 7 mer random peptide library 3 consecutive rounds of panning (Capture #1 through #3) and compared with the naïve library. For each sample the 20 top most frequent peptides are given along with the number of times they appear. The number of unique versus total peptides is shown as well. Numbers in parentheses represent the percent value of the total peptides for each category. Bold sequences indicate peptides that are carried over from Capture #1. Bold and Italic sequences indicate peptides carried over from Capture #2.

Of the total 183,451 peptides obtained in the sample of the naïve library, 92% were unique, and less than one percent (a total of 451 peptides) appeared in >4 copies, suggesting that the library well reflects the expectation from a naïve library. Notably, the most frequent peptide (RIRSEEL) existed in 24 copies, which is more than two order of magnitude less than the most common affinity purified peptide in Capture #1 (Table 1). Of the peptides in Capture #1 only the top three peptides were further amplified and found in the top 20 peptides of Captures #2 and #3. Of the 17 remaining peptides 1 can be found in the 4,824 unique peptides of Capture #3. This illustrates that most of the peptides sampled in Capture #1 are non-specific background “laced” here and there with peptides that are genuinely affinity-captured by GV4H3. However, even after a single round of amplification the situation is markedly different. Of the top 20 peptides of Capture #2, twelve are also among the top 20 found for Capture #3 (all of the remaining 8 are found within the top 100 peptides of Capture #3). Hence, there is clear evidence that the most frequent peptides obtained after Deep Panning are indeed affinity selected.

Deep sequencing the phages obtained through various steps of the experiment illustrates the trend for affinity selection and amplification of phages at the expense of marked reduction of the complexity of the random peptides present in the naïve library. As is shown in Figure S2 the vast majority of peptides are unique in the naïve library where the total number of peptide copies derived from the 20 most frequent peptides comprise an insignificant proportion (<0.15%). After two rounds of biopanning the top 20 peptides represent 74% of the 118,548 peptides sequenced while the total percent of unique copies drops to 4%. Thus Deep Panning provides a quantitative depiction of the bio-panning process and enrichment of affinity selected phages through serial rounds of panning.

### Epitope Mapping

Epitope mapping is based on the hypothesis that the peptides affinity-selected via panning reflect the structure of the epitope bound by the antibody being scanned [31]. B-cell epitopes are typically conformational and discontinuous, comprised of some 15–20 contact residues harbored within 2–3 segments of the antigen brought together via folding [32]. Clearly a 7 mer peptide cannot be expected to represent an epitope, nor must it correspond to linear segments of the antigen for recognition. Rather the panel of affinity selected peptides *collectively* represents the epitope and can be used as a dataset for computational algorithms designed to predict conformational B-cell epitopes.

**Figure 3:**
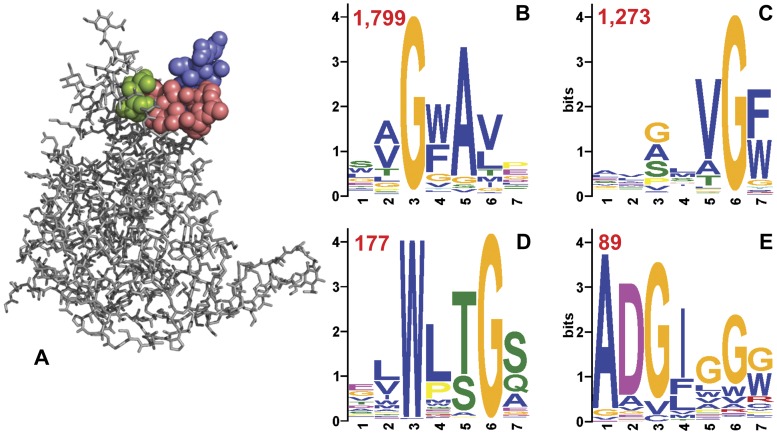
Deep Panning with mAb GV4H3. (**A**) Mapitope prediction of the GV4H3 epitope on HIV gp120. The top 20 peptides of Capture #2 (see Table 1) were used as the dataset for Mapitope prediction of the GV4H3 epitope. The single predicted cluster comprises two discontinuous segments of the antigen (green and blue) brought to flank the core of the epitope (residues 221–226, pink). (**B-E**) MEME analysis of the GV4H3 derived peptides. The 20 top most frequent peptides of Capture #3 (see Table 1) generated a major motif “AGWAV”. This motif (**B**) and three additional motifs are identified when all 4,823 peptides are analyzed. The “VGF” motif (**C**) is a simpler version of the major motif. The two additional minor motifs (**D** and **E**) do not have obvious similarity to the epitope of the mAb. The “ADGIGGG” motif clearly corresponds with the most frequent peptide ADGIVGW (see text). The numbers in red represent the number of unique peptides that define each motif.

Mapitope is such a predictive algorithm [31], which identifies significant amino acid pairs present in the phage displayed peptides that have been affinity enriched by the antibody used to pan the random peptide library. These pairs are then used to identify surface accessible residue clusters on the antigen, in this case HIV-1 gp120. These clusters are predicted to be the corresponding epitope of the antibody being studied. The output of the algorithm is a ranked list of the 5 best predictions based on the panel of peptides. As is illustrated in Figure 3A, the top 20 peptides of Capture #2 predict one single cluster that coincides precisely with the GV4H3 epitope. Success in predicting the correct epitope supports the conclusion that the peptides most amplified are indeed the product of antibody driven affinity selection and amplification.

### Motif Analysis–multiple Patterns of mAb Recognition

A total of 4,823 unique peptides were obtained in Capture #3 (Table 1). In order to determine if these represent different patterns of affinity recognition by mAb GV4H3, the motif search algorithm, MEME [33] was applied to the entire dataset. Four clear motifs were identified as are shown in Figure 3B–E. The two main motifs (Figure 3B–C) are clearly related to the main core of the GV4H3 epitope. In addition, two minor motifs (Figure 3D–E) are also found. Interestingly, the weakest motif (ADGIGGG) is actually the closest to the most amplified peptide of the experiment (ADGIVGW), thus illustrating that the most frequent and enriched peptide does not necessarily correspond best to the bona fide epitope of the antigen but rather may compliment the paratope of mAb most efficiently.

### Deep Panning HIV+ Polyclonal Serum

The situation for polyclonal serum is markedly more complex when compared with mAb analyses. Polyclonal serum is a composite of numerous mAbs, some of which may have a common target; such as a specific pathogen or epitope, while others may be totally unrelated. Each antibody binds its own set of peptides contributing to an extensive mixture of peptides representing the ensemble of antibodies active in the serum sample. Hence the profile of peptides isolated can be extremely diverse and complicated. In order to simplify matters three consecutive rounds of biopanning were performed before deep sequencing, so to reduce the amount of irrelevant background considerably.

The phage display 7 mer library was used to bio-pan a sample of purified human IgG obtained from HIV-1+ individuals (HIVIG, Nabi, Inc. Rockville, MD). After the three rounds of biopanning against the HIVIG a total of 163,400 peptides were obtained of which 7,799 were unique sequences. The question is can one identify any correspondence between the most frequent peptides and HIV? Is a pathogen related response recognizable in analyzing the peptide sequences obtained? Therefore, we asked whether or not any of these peptides could be aligned by a BLASTP analysis against HIV-1_HXB2_ gp160 so to indicate some HIV specificity.

**Figure 4:**
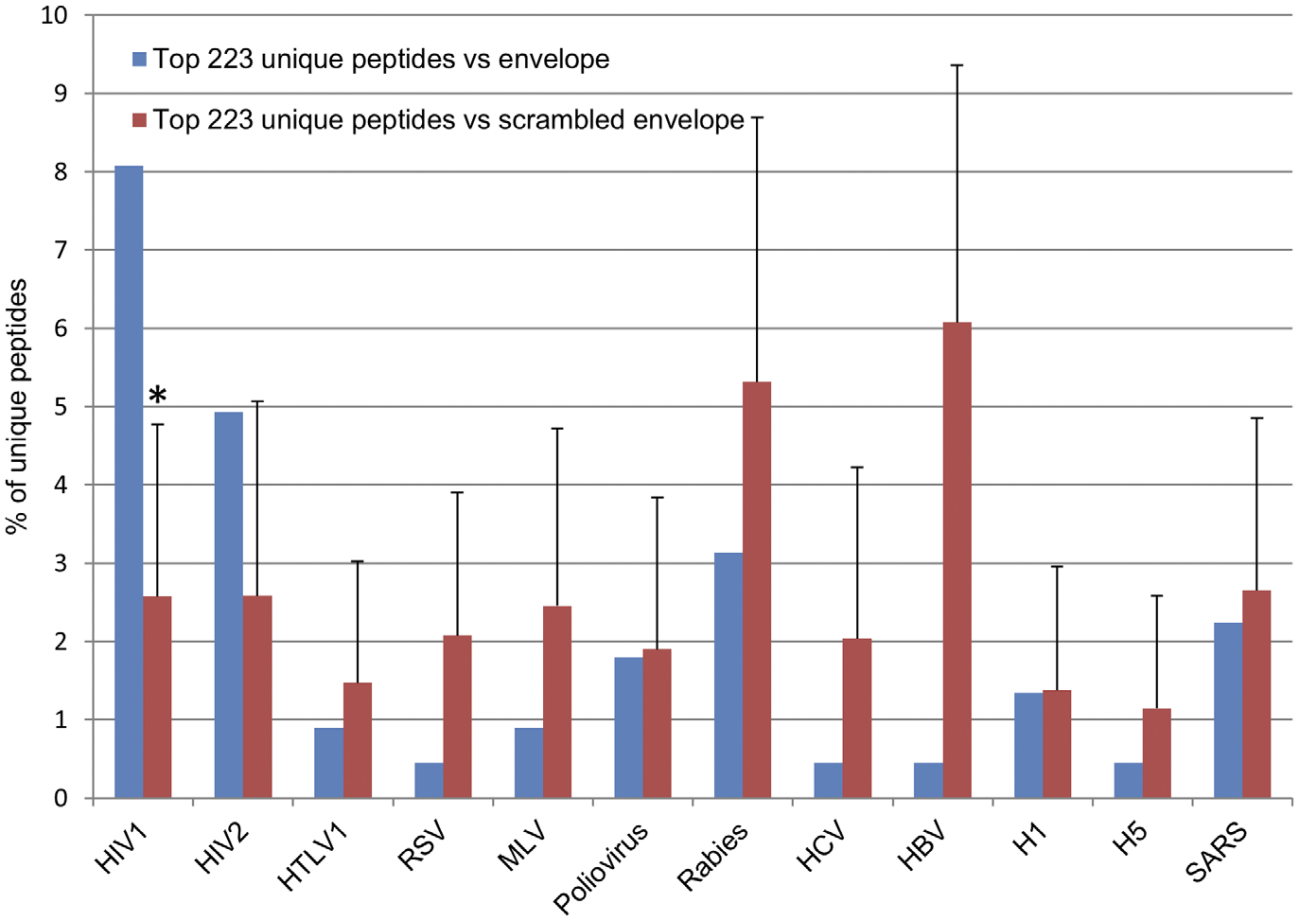
BLASTP analysis of HIVIG-captured peptides against viral coat proteins. Of the 223 top unique HIVIG-captured peptides, 18 (8%) scored hits in BLASTP analysis against the HIV-1_HXB2_ gp160 (blue). Repeating this procedure with the same protein but scrambled gives an average value of 5.5 hits when performed 1,000 times (2.5%±2.2 s.d., red). The differences between native and scrambled coat proteins BLASTP results of 11 other RNA viruses were not found to be significant. *P<0.01.

As is illustrated in Figure 4, 18 peptides (8%) of the top 223 peptides (all the peptides that were ≥5 copies) could in fact be aligned to HIV gp160. In order to evaluate if this is a significant finding the same alignment was performed against 1,000 different scrambled gp160 sequences generating an average of 2.5% ±*2.2 (s.d.)* hits which is statistically distinct from the success when using the native HXB2 sequence (Z-score = 2.5, p = 0.006). This further substantiates the hypothesis that the HIVIG-captured peptides truly represent regions of the viral gp160. Furthermore, identical analyses were conducted using the spike proteins of eleven other RNA viruses. The results reveal that there is no significant similarity between the peptides and the other viral proteins (Figure 4).

**Figure 5:**
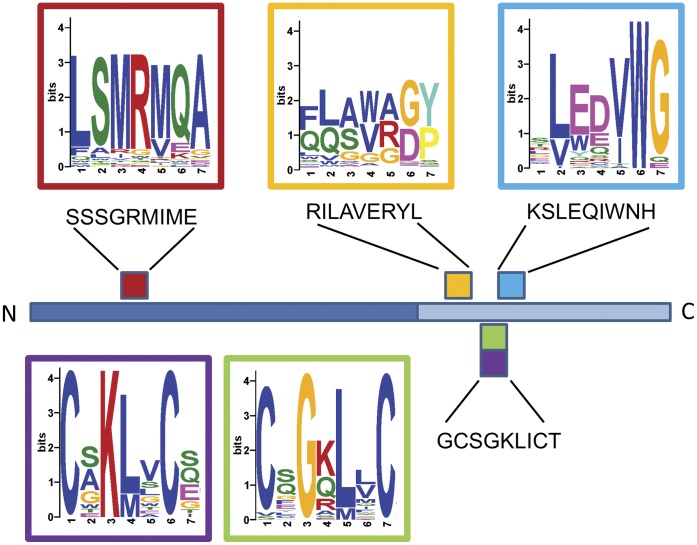
Assignment of MEME motifs within HIV gp160. The 18 BLASTP hits found within HIV-1 gp160 (see text) are located at distinct sites (indicated sequences), one within gp120 (dark blue) and 3 within gp41 (light blue) (red, yellow, cyan, green and purple squares). The genuine HIV sequences correspond to 5 of the MEME defined motifs discovered when all 648 peptides are analyzed using this algorithm.

MEME analysis on all the peptides ≥2 copies (648 unique peptides) identified 10 distinct motifs, each based on 14–162 unique peptides. As is illustrated in Figure 5, all 18 hits in the previous BLASTP analysis could be ascribed to 5 of the 10 motifs defined. This result indicates that each of the 18 peptides was not selected by accidental alignment, but rather is part of a true motif together with many similar peptides, all selected due to their correspondence to the same linear segments of the gp160 envelope protein. This result clearly illustrates that the Deep Panning of polyclonal serum produces families of affinity selected peptides that define disease related motifs that can reveal meaningful epitopes of the pathogen. This can have application towards the development of diagnostics and vaccines as is discussed below.

## Discussion

Deep Panning is shown to be an effective means to sequence and categorize panels of hundreds of thousands of affinity selected filamentous phage displayed peptides [34]. Here we have demonstrated the compatibility of combinatorial phage displayed random peptide libraries with next generation deep sequencing. Indeed, a number of studies have previously illustrated that high throughput second generation sequencing, whether using the 454 system [3], [35], [36], [37] or that of Illumina [38], [39], [40], [41], can be extremely powerful. However, in the majority of these studies recombinant Protein 3 of filamentous phage [3], [35], [37], [38], [40] or the 10B [39], [41] protein in lambda phage were used. Here we described the use of the p88 system [42] in which the fth1 vector contains two copies of the major phage protein - Protein 8. Hence, two versions of Protein 8 can be expressed and assembled in the phage without the need or use of helper phages. We have used these chimeric systems expressing libraries of random peptides ranging in length from 6–12 residues and have found all to be compatible with Deep Panning. As a case in point, we describe the use of a 7 mer library. This has led to some rather surprising results for which we decided to first carefully examine the profile and distribution of random peptides in our naïve libraries before conducting antibody analyses.

Unexpected distortions and aberrations were apparent when extensive sets of peptide sequences were examined (see also [43]). “Silent” mutated phages, genotypically containing dysfunctional inserts, were found to be enriched due to the presence of UAA/UGA stop codons that lead to abortive translation of their recombinant Protein 8 and ultimate presentation of phenotypically wild type phages. The presence of the third stop codon UAG also seems to lead to positive selection of those phages that contain this codon in their inserts. The abundance of these phages leads to a distinct biased proportion of UAG containing recombinants that affects the randomness of peptides by over representation of glutamine. At least part of this distortion can be remedied by production of the phage library in bacteria containing the *supE144* suppression tRNA which markedly improved the situation, although not completely.

Despite the irregularities identified in the library used we have learned much in the application of Deep Panning for both the analyses of mAbs and polyclonal serum. In the “traditional” phage/biopan experiment tens of peptides are produced, which are too few to identify and characterize structural motifs, this is especially true when analyzing polyclonal serum. Deep Panning, however, generates comprehensive panels of hundreds of thousands of affinity selected peptides. The frequency a given peptide is isolated is a proxy of sorts for the affinity and titer of the specific antibody in the serum that binds it. Moreover, due to the very large dataset one is able to identify structural motifs recognized by the antibodies. The quality and strength of a motif stems directly from the ability to accumulate hundreds of variant member-peptides indicating the motif’s conserved-hallmarks vs positions where variation is permissible.

Deep Panning provides an effective means to probe and characterize the IgOme. As improved libraries are constructed and more efficient computational tools are devised, detailed profiles of the repertoire of antibody specificities will become available. Peptide profiles will be surveyed for pathogen defining motifs which will serve as markers in diagnostic tests. Moreover, it has been reported that the evolution of antibody specificities in HIV patients correlates with disease progression [44], [45], [46], the ability to follow this maturation of the humoral response will enable us to establish not only the presence of the infection but also the stage of disease (acute vs chronic for example).

Obviously, the intent to conduct comprehensive comparative IgOme profiles brings to question the costs of such analyses. Deep Panning is totally compatible with multiplexing multiple serum samples each tagged with its own defining DNA barcode. Hence one can expect to run dozens of serum samples in parallel on a single lane bringing costs down to very reasonable levels. The application of Deep Panning will increase basic understanding of how B-cells respond to infection or participate in immune surveillance. IgOmic profiles will provide substance and detail of the dynamics of how antibody specificities change in response to immunological insults, variations as a result of physiology and age. This will undoubtedly impact the way disease is diagnosed as well as produce new therapeutics and vaccines to treat disease and prevent it.

## Materials and Methods

### Construction of fth1-dp Vector

The fth1-dp vector is a derivative of the fth1 vector previously described [28]. To adapt the fth1 for Illumina deep sequencing, Illumina adaptors A and B were inserted upstream and downstream to the insert-flanking SfiI sites, (Figure 1 and Figure S3). Oligonucleotides corresponding to the Adaptor sequences were inserted by ‘SOEing’ PCR mutagenesis [47] using the Accuzyme polymerase (Bioline, BIO-21052). Alternatively, extended PCR primers can be used to accomplish the same as has been described previously (see for example [3], [35], [39]).

### Library Construction

Libraries were constructed as previously described [48]. For this, two 5’ biotinylated oligonucleotides were used. The first contained the redundant “library” sequence, e.g., 7×NNK flanked by BglI sites compatible with the two SfiI sites of the vector (61 bases). The second oligonucleotide, 18 bases, complemented the 3’ end of the first and was extended to “fill-in” the complementary strand using Klenow polymerase. The product was digested with BglI, the short biotinylated segments were removed with Streptavidin conjugated magnetic beads and the eluent was cloned into SfiI digested fth1-dp vector. This ligation mix was used to electroporate MC1061 or DH5alpha cells as indicated in the text.

### Biopanning

The panning procedure was carried out for the mAb GV4H3 and the polyclonal HIVIG as previously described [48]. Shortly, 6-wells tissue culture plates (Corning, 3516) were coated with protein G (Sigma-Aldrich, P4689) in Tris-buffered saline, 50 mM Tris-HCl pH 7.5, 150 mM NaCl (TBS). The wells were blocked with 0.25% gelatin in TBS (TBSG), washed briefly twice with TBS, then incubated for 4 hours with the ligand - either HIVIG (350 µg) or mAb GV4H3 (100 µg) dissolved in 1 ml of blocking solution. Unbound ligand was washed out, and the plate was incubated overnight at 4°C with 10^11^ phages of the 7 mer random peptide library suspended in 700 µl TBSG. Subsequently, the plate was washed and the bound phages were eluted in pH 2.2 and neutralized in pH 9.1 (Capture #1). For Captures #2 and #3 additional rounds of amplification and biopanning were carried out. The eluted phages were then prepared for Illumina sequencing.

### Illumina Sequencing, Sample Preparation

After biopanning the eluted phages (500 µl) were precipitated with 200 µl PEG/NaCl for 2.5 h on ice at 4°C. Tubes were centrifuged at 13,000 rpm for 45 min at 4°C. The phage pellets were resuspended in 40 µl TBS and used as templates for PCR. Parallel PCR reactions (50 µl) were prepared, each containing phage template (10 µl), polymerase (Taq (Larova GmbH, VAR-04)), sense (AATGATACGGCGACCACCGAGATCTACACTCTTTCCCTACACGACGCTCT) and antisense (CAAGCAGAAGACGGCATACGAGCTCTTCCGATCT) primers corresponding to the Illumina adaptors A and B. For the analysis of the naïve libraries, four samples of 4×10^8^ phages each were amplified individually by the PCR reactions.

The thermal profile was:

1. 95°C 5 min
2. 95°C 1 min
3. 53°C 1 min
4. 72°C 20 sec
5. Go back to step 2×34
6. 72°C 5 min

The amplified PCR products were validated for size (152 bp) by running in 2% agarose gel. PCR samples were purified by RBC Real Genomics HiYield ™ Gel/PCR DNA Fragments kit (RBCBioscience, YDF100 ) into 40 µl volume. All 4 PCR cleaned products were united and their concentration was measured. The sample was dried by speed vac and sent for Illumina sequencing.

### Illumina Sequencing

The dried samples were resuspended in 20 µl of elution buffer and analyzed using an Agilent BioAnalyzer 2100 to verify their quantity and quality. Quantitation for sequencing was done using qPCR, after which samples were normalized and 5 µl to 15 µl were spiked in to 1,000 µl (1.5 pM) of the Illumina PhiX control sample.

140 µl of the normalized samples plus PhiX control were dispensed into strip tubes. These samples in strip tubes were loaded on to a flowcell and bridge amplified for approximately 4 h using a cBot and/or cluster station to obtain millions of the same copies of DNA template. The amplified template in a flowcell was then loaded on the Illumina HiSeq 2000 and/or Genome Analyzer IIx and sequenced using the Illumina sequencing by synthesis chemistry.

### Simulation Study to Characterize the Distribution of the Most Common Peptide in Naive Peptide Libraries

We estimated the expected distribution of the most frequent peptide, for naïve libraries, i.e., peptide libraries that were not subjected to any affinity selection (e.g., biopanning). Using this distribution, we were able to statistically determine if the observed number of repeats for the most common peptide can be obtained by chance alone. This random distribution was computed using a computer simulation. Specifically, we first randomly generated NNK sequences to equal the size of the random library before the PCR sampling. Next, these sequences were computationally amplified to mimic random PCR amplification. Finally, a subset of these sequences was selected to mimic sampling for sequencing. The frequency of the most common peptide in the generated library was then recorded. This process was repeated one hundred times to generate the expected distribution of the most frequent peptide. The C++ code for this simulation scheme is freely available from the authors upon request.

### Epitope Mapping

Epitope mapping was performed using the Mapitope algorithm, which is implemented in the Pepitope server (freely available at: http://pepitope.tau.ac.il/ [49]). Mapitope algorithm was described before by Bublil et al [31]. Briefly, given a set of peptides derived from a bio-panning experiment and the 3D structure model of the antigen of interest, Mapitope maps pairs of residues that are significantly overrepresented in the set of peptides onto the antigen 3D structure.

### Pre-processing of Sequence Data

The Illumina sequences were analyzed with the assistance of BioPerl packages [50]. For all analyses 100% fidelity for a barcode reference sequence residing between the adaptor A and first SfiI site (see Figure 1) was mandatory (in sequence and reading frame). For the HIVIG peptides the sequences were further filtered, and any sequence with more than 1 mismatch in the non-insert region was cleared.

### Analysis of Polyclonal Serum

BLASTP [51] was used to detect sequence similarity between the HIVIG-captured peptides and HIV-1_HXB2_ gp160 or the spike proteins of eleven other RNA viruses as shown in Fig. 4. All HIVIG-captured peptides that had more than 5 copies were considered for this analysis resulting in total of 155,206 peptides (reflecting 223 unique peptides). The viral proteins chosen for the analyses were the sequences from the NCBI RefSeq database. Their UniProtKB accession IDs are: HIV1: P04578.2; HIV2: P18094.1; HTLV: P14075.1; RSV: P03396.1; MLV: P26804.1; Poliovirus: P03300.3 (1–881); Rabies: P06747.1, P08671.1, P08667.1,;HCV: P27958.3 (192–746); HBV: Q76R62.2, Q76R62.2; Influenza H1: Q9WFX3.2; Influenza H5: Q6DQ18.1; SARS: P59594.1;.

BLASTP parameters were adjusted to deal with short peptides (7-mers). The parameters we used are as follows: E-value threshold (-e): 2,000; substitution matrix (-M): PAM30; Word-size (-W): 2 and without using composition-based statistics (-t 0). The BLASTP results were further processed such as only peptides that (1) their alignment length with the target protein is at least 5 amino acids and (2) share more than 80% sequence identity with the viral segment were considered as meaningful hits (true hits). To compare these results with the expected number of random hits, the viral amino acid sequences were scrambled and BLASTP procedure was repeated for each scrambled sequence (repeated 1,000 times). We consider the average number of matches between the scrambled proteins and the peptides set as the random background (random hits). The number of true hits was compared to the number of random hits using the Z-test and p value was calculated under the assumption that the number of random hits is normally distributed.

### Motif Analysis

We used the Multiple EM for Motif Elicitation (MEME) algorithm [33] for motif discovery in order to characterize the peptides captured by the antibodies. For MEME analysis, all unique (protein) sequences were used while assuming that each motif is expected to occur zero or one time in each sequence (zoop mode).

## Supporting Information

Figure S1 **GV4H3 validation.** The fth1-dp phage (indicated) was spotted directly onto a nitrocellulose membrane filter along with a phage containing a DNA insert corresponding to the GV4H3 epitope (AGFAIL). The membrane was subsequently immunoblotted with GV4H3 and positive clone was sequenced to validate the presence of AGFAIL insert and Illumina adaptors A and B.
(TIF)

Figure S2 **Progressive enrichment of affinity selected peptides.** 90% of the peptides in the naïve library are unique (blue) where the 20 top most frequent peptides (red) constitute 0.15%. Each round of consecutive panning leads to a drop of the total fraction of unique peptides accompanied by enrichment of those most highly affinity-selected.
(TIF)

Figure S3 **Construction of fth1-dp phage vector using ‘SOEing’ PCR.** The fth1 vector was utilized as template (A) for the generation of two independent PCR products; the first (B, red) contained a BglII site at its 5′ end and the adapter A sequence (A - orange) followed by a barcode and a SfiI site at its 3′ end (primers 2201s: GCTAGCCATCAGATCTGCACTG and 9602as: GGACGTCATTACCGGCCACGTTGGCCNCCNGANCCNGATAAGATCGGAAGAGCGTCGTGTAGGGAAAGAGTGTTGCCTTCCGCCGCAAAGCTTAAC). The second (C, purple) contained a SfiI site followed by adapter B (B - blue) at its 5′ end and an EagI site at its 3′end (primers 9301s: GGCCGGTAATGACGTCCATAATGGCCTCTGGGGCCCAGATCGGAAGAGCTCGTATGCCGTCTTCTGCTTCGGACCCTGCGAAGGCAGCATTCG and 2301as: AAACAGCGGCCGCTATCAACTGG). The primers were mixed and further amplified (D) to generate a single product (E) which was double digested with BglII and EagI and inserted into a fth1 digested with these two enzymes to generate the fth1-dp phage vector (F). The ligated vectors were used to transform MC1061 cells by electroporation, colonies were picked and validated for sequence correctness.
(TIF)
