# Supplementary figures and images for: Deep Panning: Steps towards Probing the IgOme
Source: PLoS One. 2012 Aug 1;7(8):e41469. doi: 10.1371/journal.pone.0041469 (PMC3409857; doi:10.1371/journal.pone.0041469)

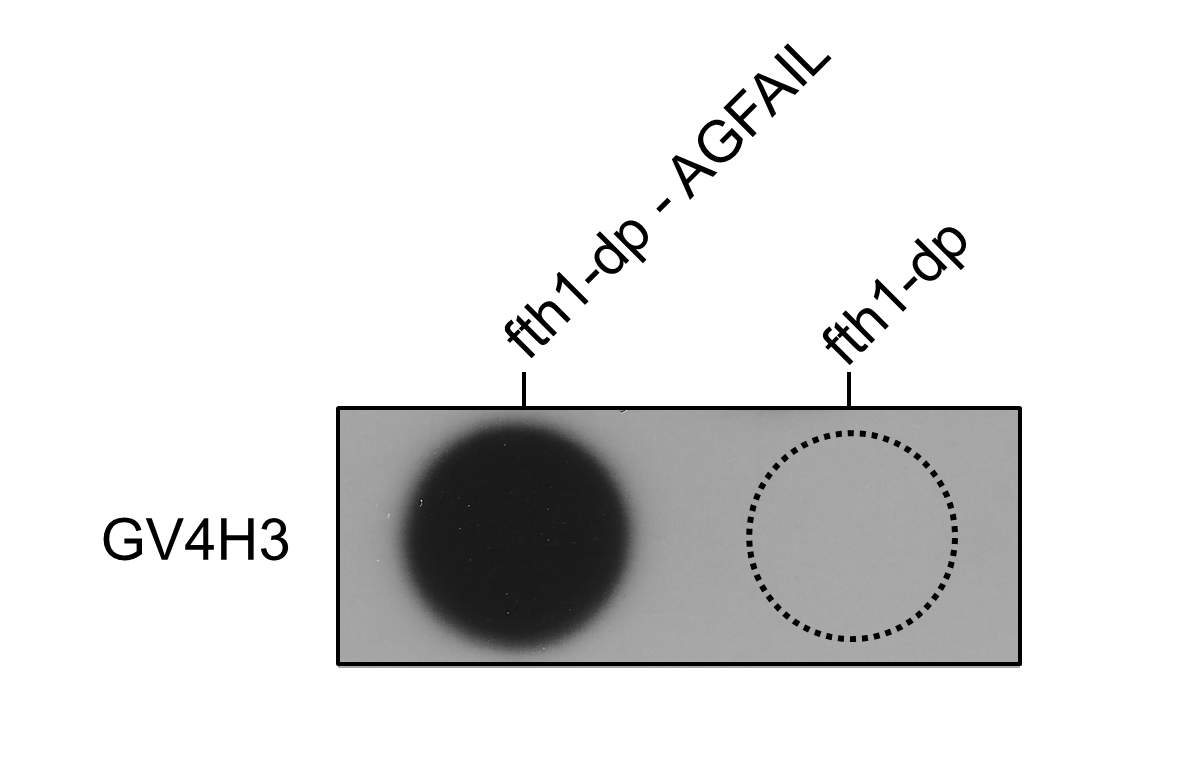

Supplement: Figure S1 — GV4H3 validation. The fth1-dp phage (indicated) was spotted directly onto a nitrocellulose membrane filter along with a phage containing a DNA insert corresponding to the GV4H3 epitope (AGFAIL). The membrane was subsequently immunoblotted with GV4H3 and positive clone was sequenced to validate the presence of AGFAIL insert and Illumina adaptors A and B. (TIF) [file pone.0041469.s001.tif]

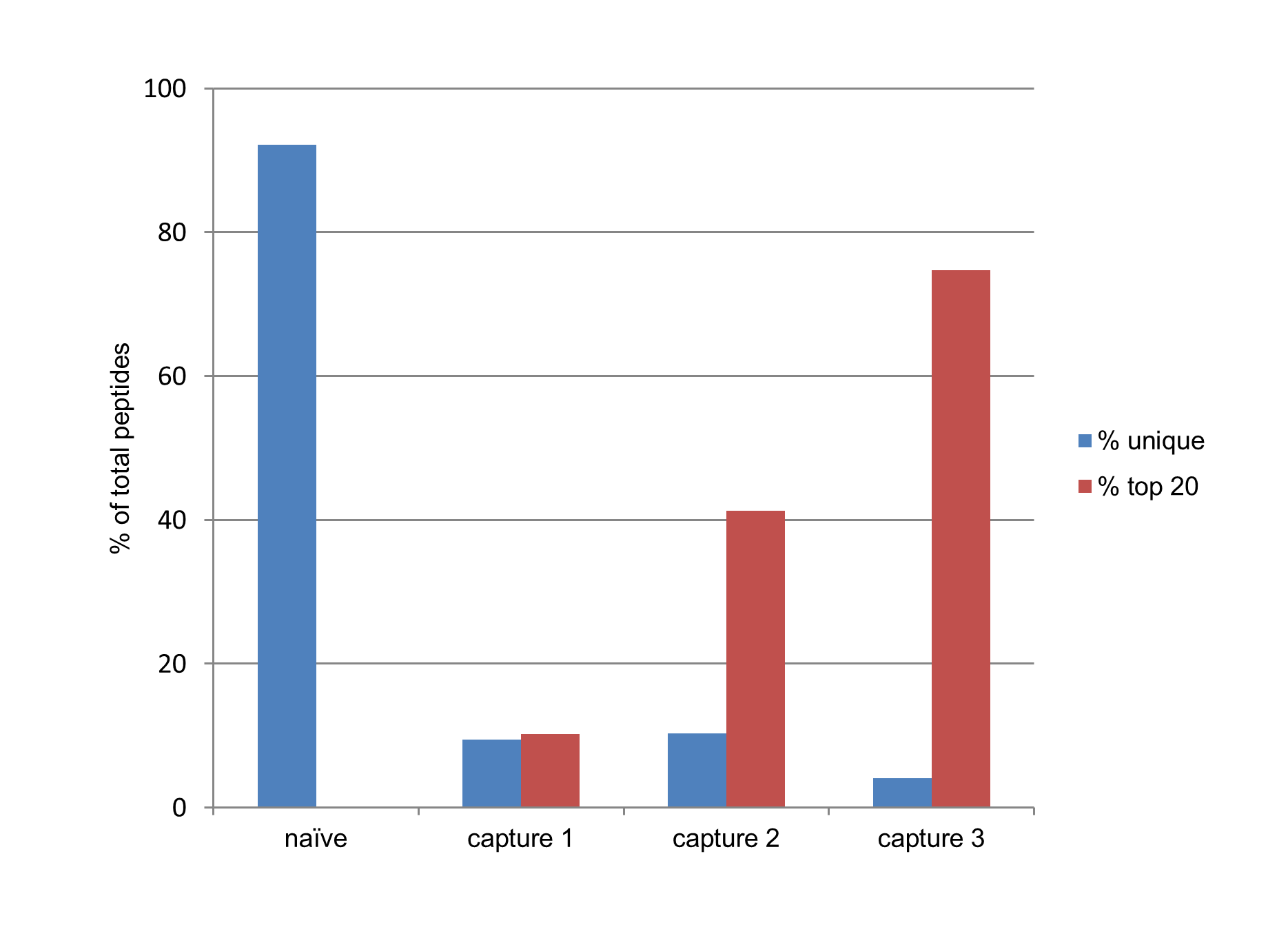

Supplement: Figure S2 — Progressive enrichment of affinity selected peptides. 90% of the peptides in the naïve library are unique (blue) where the 20 top most frequent peptides (red) constitute 0.15%. Each round of consecutive panning leads to a drop of the total fraction of unique peptides accompanied by enrichment of those most highly affinity-selected. (TIF) [file pone.0041469.s002.tif]

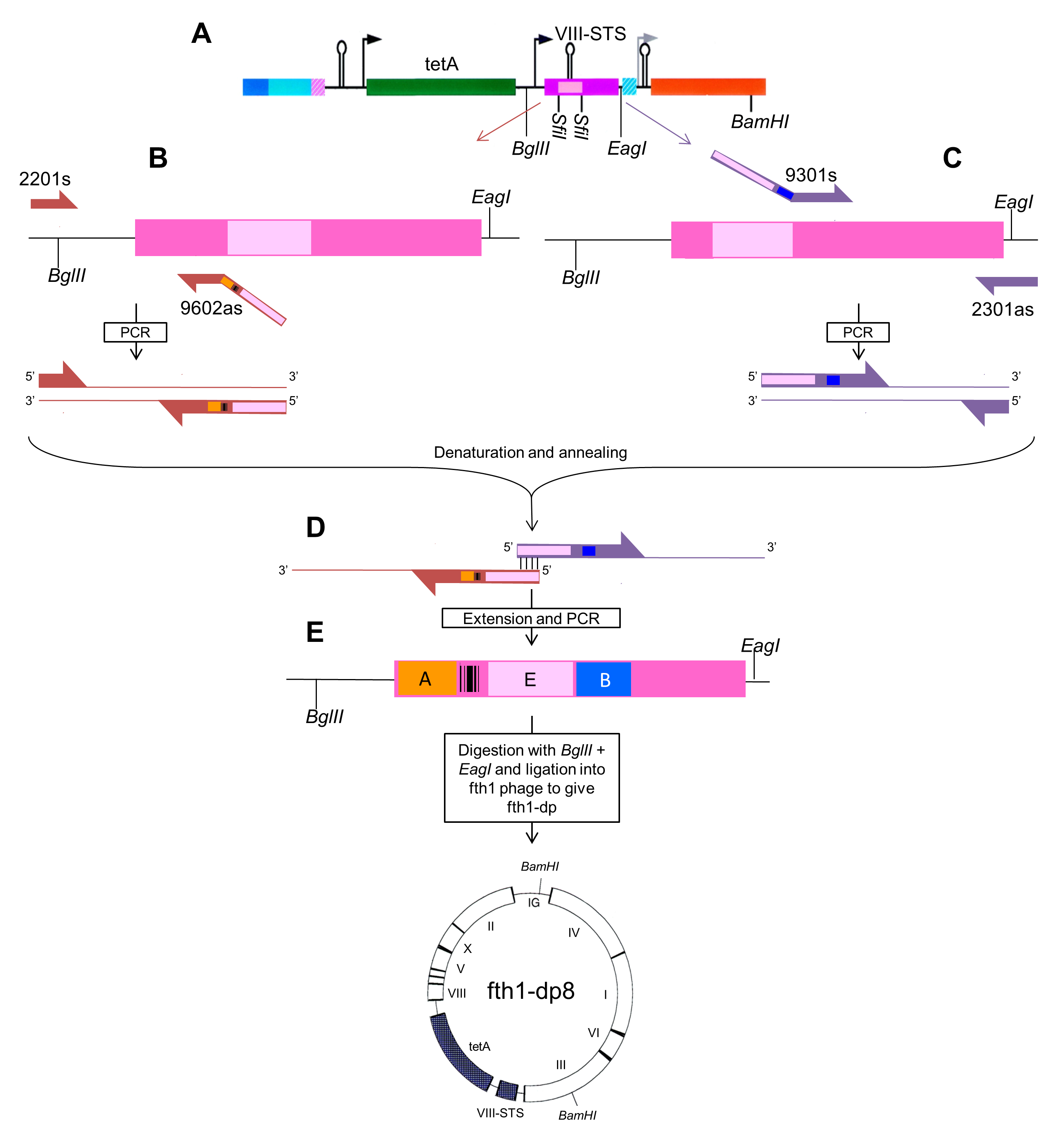

Supplement: Figure S3 — Construction of fth1-dp phage vector using ‘SOEing’ PCR. The fth1 vector was utilized as template (A) for the generation of two independent PCR products; the first (B, red) contained a BglII site at its 5′ end and the adapter A sequence (A - orange) followed by a barcode and a SfiI site at its 3′ end (primers 2201s: GCTAGCCATCAGATCTGCACTG and 9602as: GGACGTCATTACCGGCCACGTTGGCCNCCNGANCCNGATAAGATCGGAAGAGCGTCGTGTAGGGAAAGAGTGTTGCCTTCCGCCGCAAAGCTTAAC). The second (C, purple) contained a SfiI site followed by adapter B (B - blue) at its 5′ end and an EagI site at its 3′end (primers 9301s: GGCCGGTAATGACGTCCATAATGGCCTCTGGGGCCCAGATCGGAAGAGCTCGTATGCCGTCTTCTGCTTCGGACCCTGCGAAGGCAGCATTCG and 2301as: AAACAGCGGCCGCTATCAACTGG). The primers were mixed and further amplified (D) to generate a single product (E) which was double digested with BglII and EagI and inserted into a fth1 digested with these two enzymes to generate the fth1-dp phage vector (F). The ligated vectors were used to transform MC1061 cells by electroporation, colonies were picked and validated for sequence correctness. (TIF) [file pone.0041469.s003.tif]
